# Supplementary material for: DJ-1 Expression Might Serve as a Biologic Marker in Patients with Bladder Cancer
Source: Cancers (Basel). 2022 May 21;14(10):2535. doi: 10.3390/cancers14102535 (PMC9139869; doi:10.3390/cancers14102535)
Supplement: Supplementary file 1 [file cancers-14-02535-s001.zip › cancers-1703029-SI.pdf]

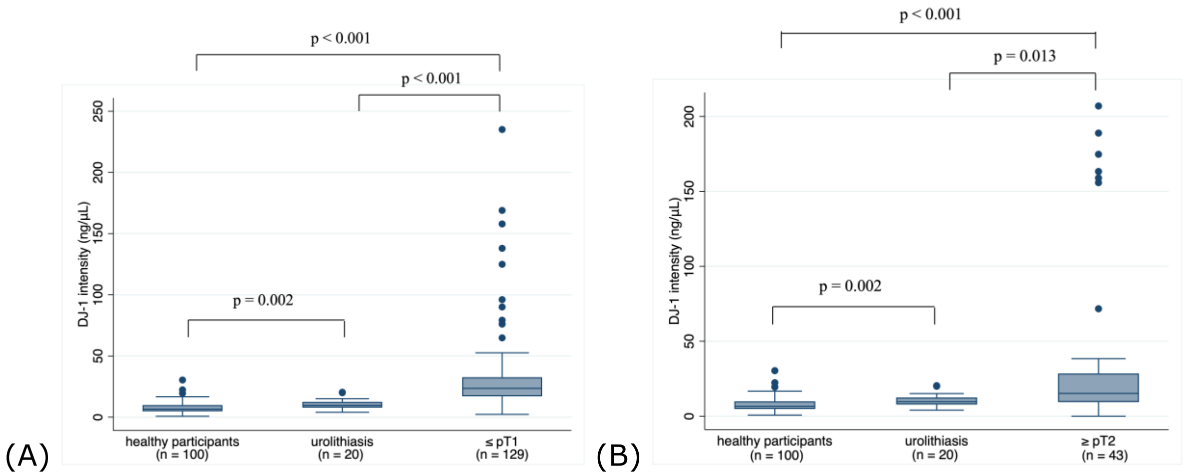

Figure S1. Serum concentrations of DJ-1 protein by reverse-phase protein array analysis (**A**) in patients with non-muscle-invasive bladder cancer or with urolithiasis and in healthy participants, and (**B**) in patients with muscle-invasive bladder cancer or with urolithiasis and in healthy participants.

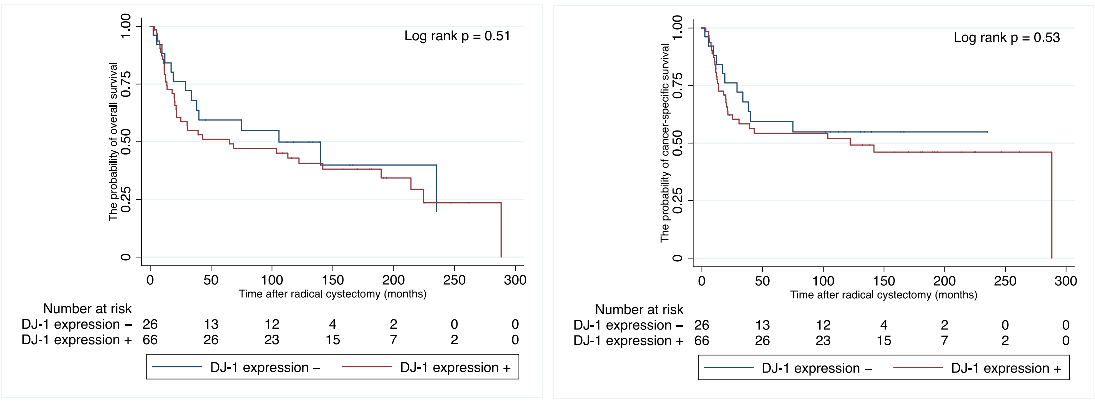

Figure S2. Probability of overall survival and cancer-specific survival after radical cystectomy based on positive and negative DJ-1 expression in urothelial carcinoma.

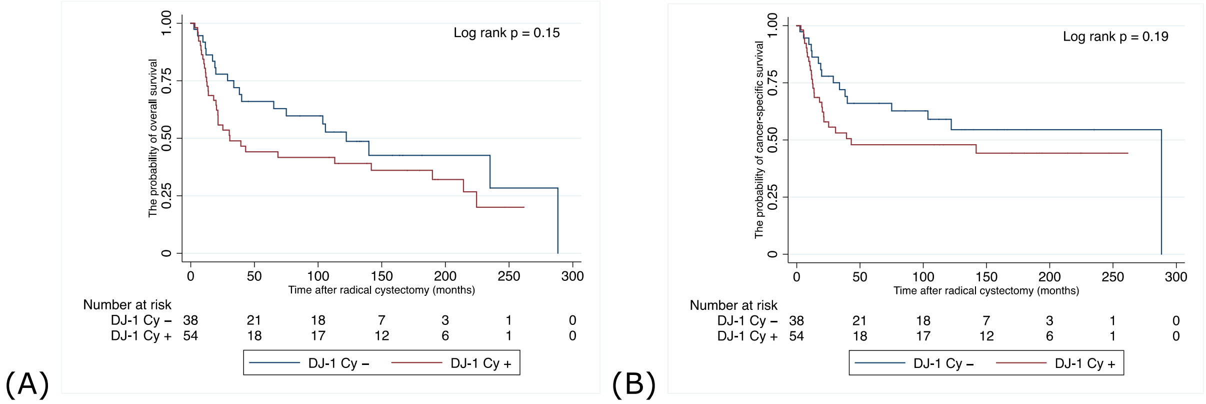

Figure S3. Probability of (A) overall survival and (B) cancer-specific survival after radical cystectomy based on positive and negative DJ-1 expression in cytoplasm.

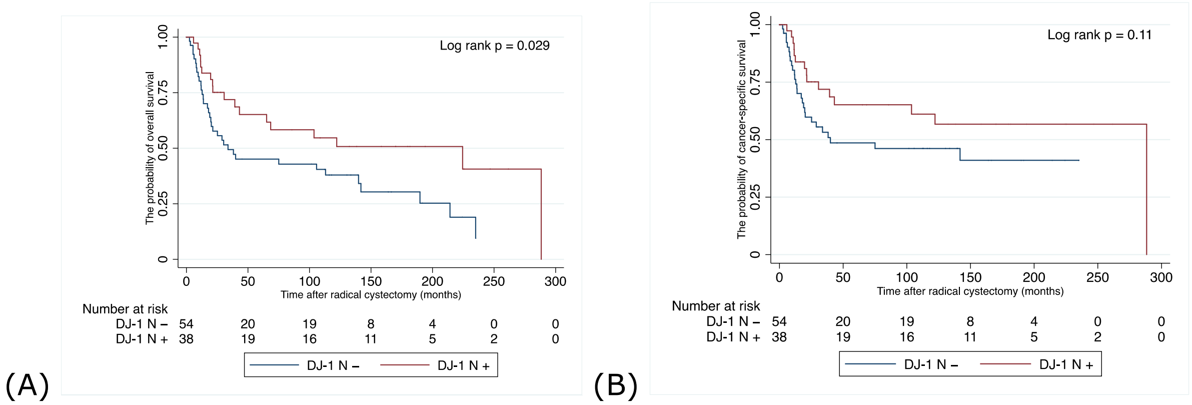

Figure S4. Probability of (A) overall survival and (B) cancer-specific survival after radical cystectomy based on positive and negative DJ-1 expression in the nucleus.
